# Human Factor H and anti-Neisserial surface protein A (NspA) antibodies compete for overlapping binding sites on meningococcal NspA

**DOI:** 10.1128/iai.00339-24

**Published:** 2025-02-24

**Authors:** Dhaarini Raghunathan, Susie Sohee Lim, Gregory R. Moe, Peter T. Beernink

**Affiliations:** 1 Division of Infectious Diseases and Global Health, Department of Pediatrics, School of Medicine, University of California 12224 , San Francisco, California, USA; 2 OMVax, Inc., San Francicso, CA; Universite de Geneve, Genève, Switzerland

**Keywords:** antigen, complement, Factor H, gonococcus, meningococcus, *Neisseria*, NspA, surface proteins

## Abstract

Neisserial surface protein A (NspA) is a small, conserved outer membrane protein that has been investigated as a vaccine antigen against meningococcal disease. After NspA had been tested in humans, this antigen was discovered to recruit the human complement regulator Factor H (FH). Previous studies in transgenic mice showed that human FH decreased the protective antibody responses to NspA. The purpose of the present study was to map the binding sites for human FH and anti-NspA antibodies. We found that an anti-NspA monoclonal antibody (mAb), AL-12, inhibits binding of FH to NspA by enzyme-linked immunosorbent assay (ELISA). Based on this result, we tested the roles of the 10 charged residues on the external loops of NspA in binding these two molecules by site-specific mutagenesis and binding experiments. Through ELISA and surface plasmon resonance experiments, we show that three aspartate (D) residues, D77 on loop 2 and D113 and D118 on loop 3, are important for binding human FH. Further, residues D113 and D118, as well as lysine 79 and arginine 109, are involved in binding mAb AL-12, which binds to a conformational epitope. The results have implications for strategies to increase NspA immunogenicity by decreasing binding to human FH, as has been done with other antigens that recruit this complement regulator.

## INTRODUCTION

*Neisseria meningitidis and N. gonorrhoeae* are human-specific pathogens that are the causative agents of invasive meningococcal disease and the sexually transmitted infection gonorrhea, respectively. Meningococcal infections result in significant morbidity and mortality, and vaccines are available for prevention of disease caused by five of the pathogenic capsular groups (1). Gonococcal infections are more prevalent and have become a greater health concern since the emergence of antimicrobial resistant strains (2, 3). Although no vaccine has been licensed to protect against *N. gonorrhoeae* (4), vaccines that contain detergent-extracted meningococcal outer membrane vesicles (OMV) to prevent disease caused by meningococcal group B (MenB) strains have been shown to elicit partial protection against gonorrhea in retrospective studies (5, 6).

Neisserial surface protein A (NspA) is a small, surface-exposed integral membrane protein that is highly conserved among meningococcal strains and, therefore, was proposed as a vaccine candidate (7). NspA was predicted to adopt a beta-barrel structure with eight antiparallel beta strands and four surface-exposed connecting loops (8), which was later confirmed by X-ray crystallography (9). Recombinant NspA, which was administered to mice with Quil A as an adjuvant, induced bactericidal serum antibodies and protected the animals from challenge with live MenB bacteria (7). NspA was ultimately tested in humans, but the formulations contained unfolded NspA and detergents and did not elicit bactericidal antibodies against a MenB strain with a matched NspA sequence (10).

Many pathogens recruit the soluble host complement regulators Factor H (FH) and C4b-binding protein (C4BP) as an immune evasion mechanism to protect themselves from complement-mediated killing (11, 12). *N. meningitidis and N. gonorrhoeae* express virulence factors that are specific for the human host to promote pathogenesis and evade the immune system. NspA is one such virulence factor that binds to human FH and FH-like protein-1 (FHL-1) and contributes to complement resistance (13, 14). The bactericidal antibody responses of transgenic mice that expressed human FH were lower than those of wild-type (WT) mice lacking human FH (15). Therefore, mutating NspA to decrease the binding of human FH is a potential strategy to increase the bactericidal antibody responses to NspA, which is highly conserved between *N. meningitidis* and *N. gonorrhoeae*. Despite the overall similarity between meningococcal and gonococcal NspA, amino acid sequence differences in loops 2 and 3 modulated FH binding (14), which suggest regions of NspA for further mapping studies.

Murine monoclonal antibodies (mAbs) have been generated against NspA expressed in native OMV (NOMV), which are naturally released from the outer membranes of gram-negative bacteria. Murine mAb Me-1, which was raised against an outer membrane preparation extracted with lithium chloride from a MenB strain, reacted in dot immunoblots with 248 of 250 meningococcal strains tested (7). This mAb mediated bactericidal activity against several MenB strains and passively protected mice against parenteral challenge with the bacteria (7). Another anti-NspA mAb designated Me-7 mediated bactericidal activity against most of the MenB strains tested (16). Sequence differences in the extracellular loops of NspA from the resistant group A strain MCH88 were proposed to explain the lack of reactivity and functional activity of mAb against this strain. Two other anti-NspA mAbs, AL-12 and 14C7, were raised against NspA expressed in *Escherichia coli* or *N. meningitidis* NOMV, respectively (17). Like mAb Me-7, these two mAbs reacted with NspA from the strains used as the immunogens, but not with NspA from strain MCH88 (18). Multiple sequence alignments of NspA from strains that were reactive or nonreactive with these mAbs suggested that residues in loops 2 and/or 3 were involved in the epitopes, which was confirmed by site-specific mutagenesis of several of the polymorphic residues (18). Both mAbs AL-12 and 14C7 elicited bactericidal activity against several MenB strains with sufficient expression of an NspA sequence that reacted with the mAbs (17). These mAbs also conferred passive protection on infant rats against MenB challenge (17, 19).

Based on the decreased immunogenicity of NspA in human FH transgenic mice (15), a mutant NspA antigen with decreased FH binding that retained protective epitopes would represent an improved vaccine antigen for humans. Therefore, the objective of the present study was to use a targeted mutagenesis approach to better understand the role of specific NspA residues in their interactions with human FH and anti-NspA mAbs. We selected charged residues for mutagenesis based on the hypothesis that salt bridges and/or charged hydrogen bonds contribute binding energy to protein–protein interactions. We systematically changed each of the 10 charged residues in the four extracellular loops of NspA to alanine (A). We tested the NspA mutants for binding of human FH and an anti-NspA mAb and found that some of the same residues were important for binding both molecules.

## RESULTS

### Detection of meningococcal NspA in the outer membrane of *E. coli*

**Fig 1:**
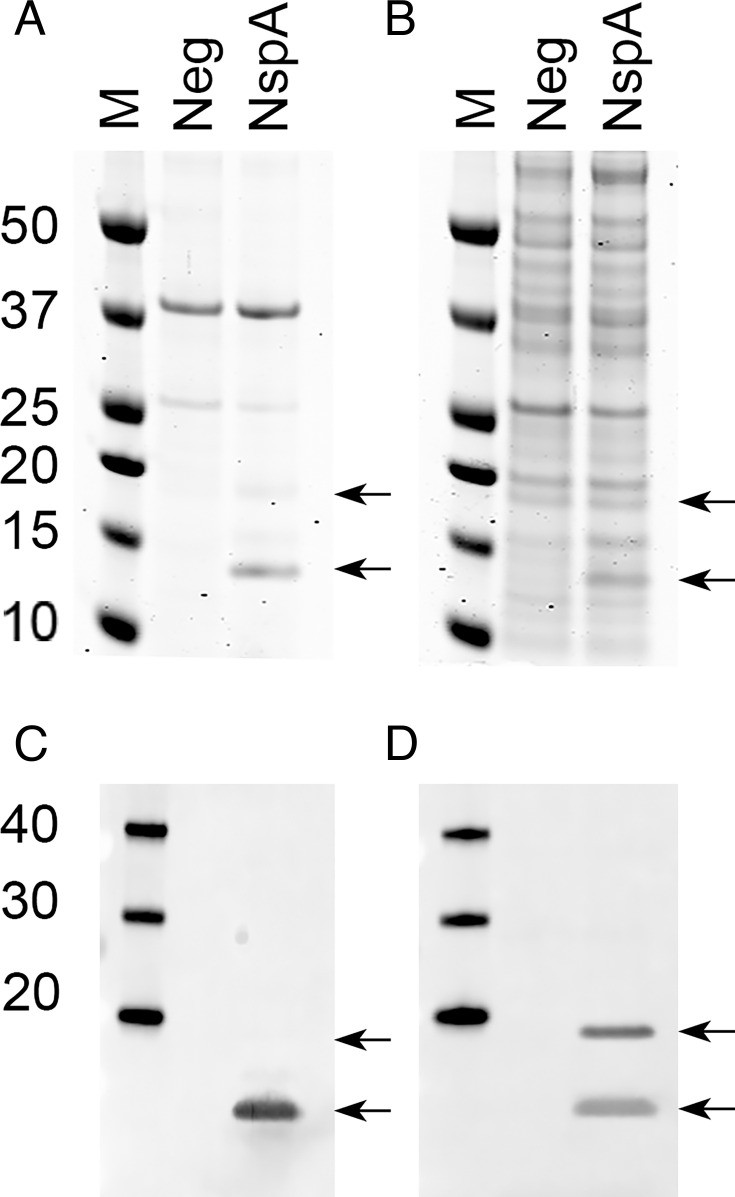
Detection of meningococcal NspA expressed in *E. coli*. (A) Expression of NspA in *E. coli* NOMV detected by SDS-PAGE and Coomassie blue staining. M, molecular weight marker; Neg, *E. coli* BL21(DE3) containing the pUC19 parent plasmid lacking the NspA gene; NspA, *E. coli* BL21(DE3) expressing wild-type NspA. The two arrows indicate different species of NspA (see text). (B) Expression of NspA in whole *E. coli* lysates detected by SDS-PAGE and Coomassie blue staining. (C) Western blot of whole *E. coli* lysates detected with mouse anti-NspA mAb AL-12. (D) Western blot probed with mouse polyclonal anti-NspA antibodies. The lane designations for panels B–D are the same as for panel A.

Recombinant meningococcal NspA from strain Su1/06 (15) was expressed with its signal sequence in *E. coli* NOMV, which were present in culture supernatants. Two NspA species with different mobilities that were absent in NOMV from *E. coli* transformed with a negative control plasmid were detected by sodium dodecyl sulfate polyacrylamide gel electrophoresis (SDS-PAGE) (Fig. 1A). The slower migrating band had an apparent molecular weight of ~18 kDa (indicated by the upper arrow), which was consistent with that calculated from the amino acid sequence, 16.5 kDa. The faster migrating species had an apparent mass of ~13 kDa (indicated by lower arrow). A similar pattern was seen for whole *E. coli* lysates (Fig. 1B), although the presence of additional cytosolic proteins made it more difficult to distinguish NspA from other proteins. By Western blotting of whole *E. coli* lysates, only the faster migrating species was detected with the anti-NspA mAb AL-12, (Fig. 1C). Since AL-12 recognizes a conformational epitope of NspA (18), we infer that a fraction of NspA likely refolds after denaturation and before electrophoresis, and the species recognized by AL-12 corresponds to refolded NspA. Both species were detected with polyclonal anti-NspA antibodies on a duplicate blot (Fig. 1D). The polyclonal antibodies were raised against unfolded NspA, and it is likely that these antibodies recognize both unfolded and refolded NspA.

### Inhibition of human FH binding by mAb AL-12

**Fig 2:**
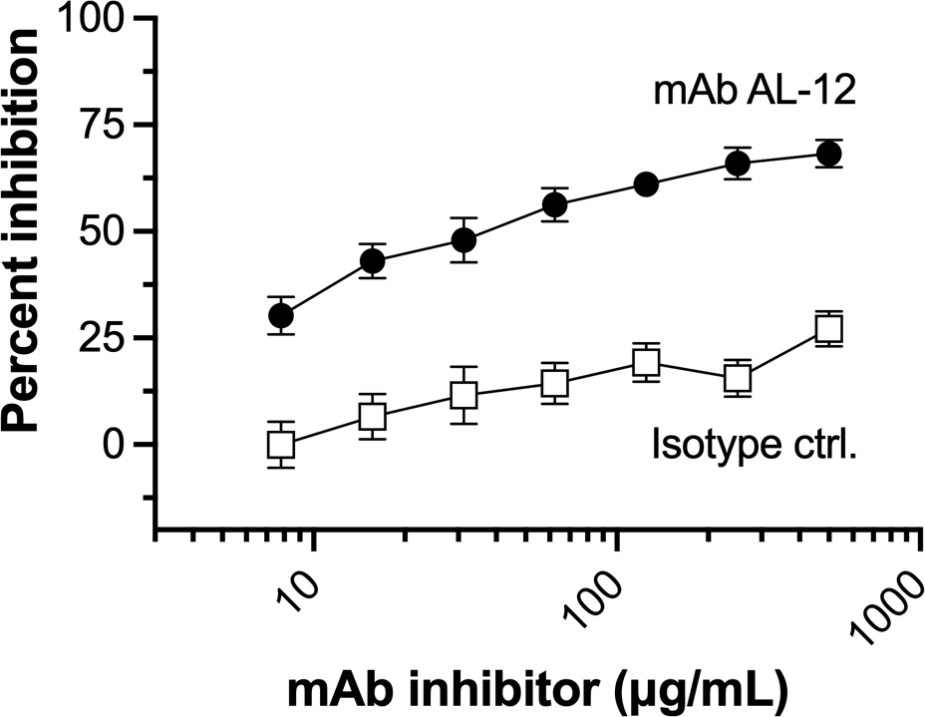
Inhibition of binding of FH to refolded WT NspA by mAb AL-12. Varying concentrations of anti-NspA mAb AL-12 were used to inhibit binding of a fixed concentration of human FH to purified, refolded NspA by ELISA. Filled circles, mAb AL-12; open squares, isotype control mAb JAR 4. The experiment was performed in triplicate, and the symbols and error bars represent mean ± SE.

As described in the *Introduction*, a previous study used natural polymorphisms in NspA among meningococcal strains to identify amino acid residues in loops 2 and 3 that were important for binding anti-NspA mAbs (18). In addition, polymorphisms between meningococcal and gonococcal NspA were used to determine that these loop regions were also important for binding human FH (14). Therefore, we tested the ability of different concentrations of mAb AL-12 to inhibit binding of a fixed concentration of human FH to purified, refolded NspA by enzyme-linked immunosorbent assay (ELISA). mAb AL-12 inhibited binding of human FH by approximately 70% at 500 µg/mL (Fig. 2). In contrast, an isotype-matched negative control mAb, JAR 4 (20) gave <25% inhibition at the same concentration. This experiment provided direct evidence that human FH and a functionally active (i.e., bactericidal) mAb compete for overlapping binding sites on NspA.

### Rationale for construction of site-specific NspA mutants

**Fig 3:**
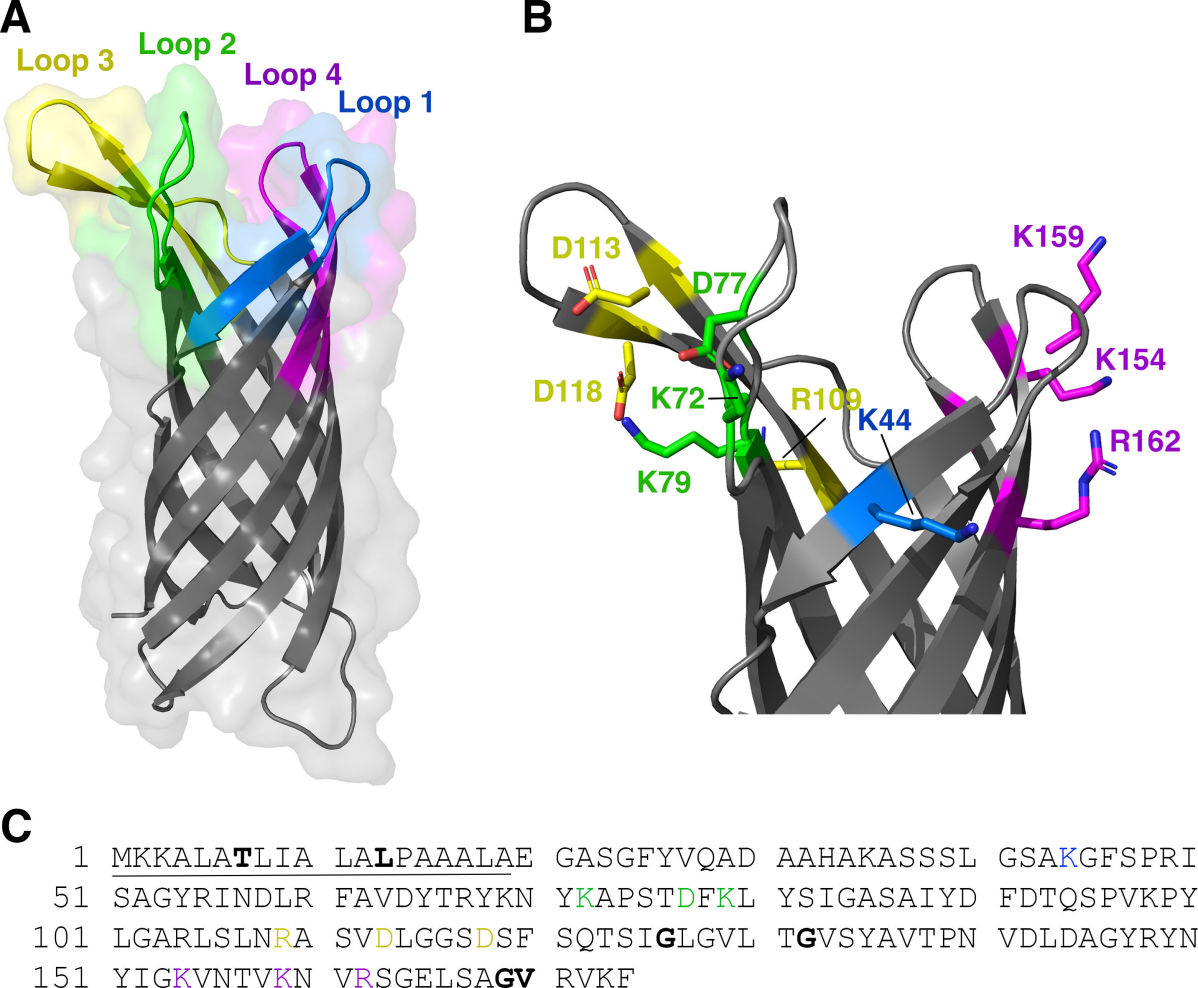
Crystal structure and sequence of NspA showing sites of amino acid substitutions. (A) Crystal structure of NspA [PDB ID 1P4T (9)], showing its four external loops, which contain the binding site for human complement FH and the epitopes recognized by anti-NspA antibodies. Loops 1 through 4, as defined previously (8), are shown in color. (B) Closer view of loops 1 through 4 showing side chains of charged residues targeted for mutagenesis in stick representation. (C) Amino acid sequence of NspA from strain Su1/06. The signal sequence is underlined, and the residues targeted for mutagenesis are shown in color. Sites of natural variation (8) are shown in bold. Panels A and B prepared with PyMol (The PyMOL Molecular Graphics System, Version 3.0 Schrödinger, LLC).

The approaches previously used to map the binding sites for human FH and anti-NspA mAbs based on sequence polymorphisms would not have detected conserved residues involved in binding human FH or anti-NspA mAbs. We took a non-biased approach by mutagenesis of each of the charged residues in all four of the extracellular loops of NspA. Our hypothesis is that charged residues are likely to contribute important binding interactions through salt-bridges and charged hydrogen bonds. Based on the extracellular loops 1 through 4 defined previously (8) and the known crystal structure of NspA (9) (Fig. 3A), we substituted each of the 10 charged amino acid residues in these loops with A (Fig. 3B and C). These include two positions that were at or near the sites of natural amino acid variation between meningococci and gonococci, K72 and D113 (14, 18).

### Effects of charged amino acid substitutions on FH binding to NspA

**Fig 4:**
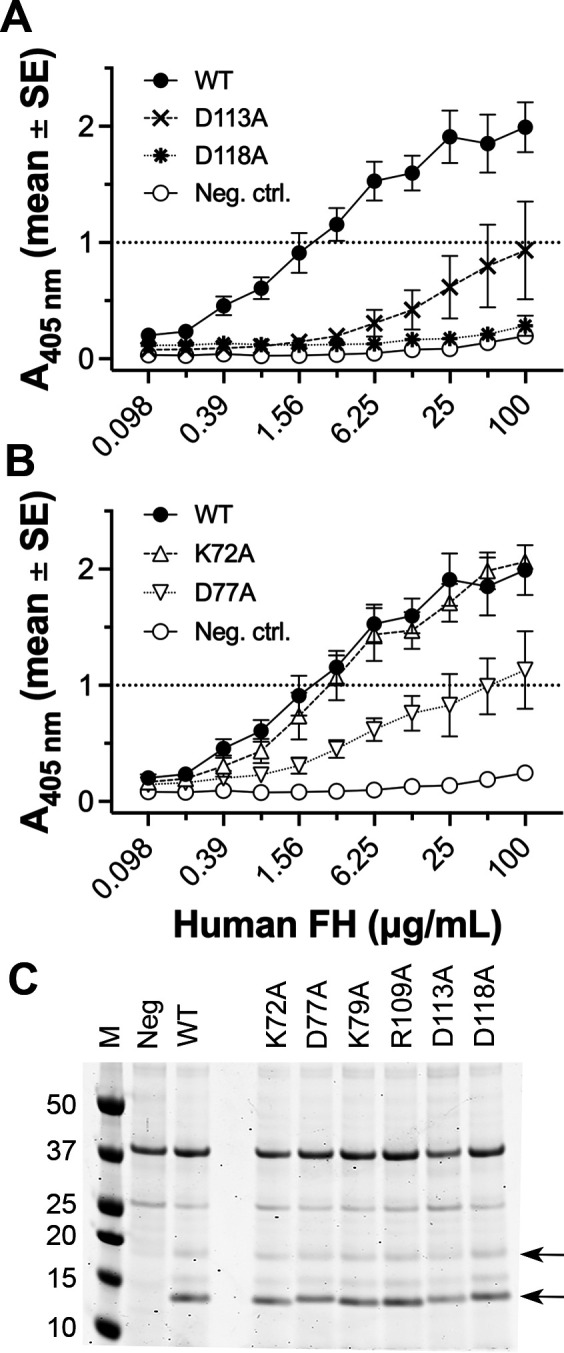
Binding of human FH to NspA loop mutants. (A) Human FH binding to NspA loop 3 mutants expressed in whole *E. coli* bacteria measured by ELISA. (B) Human FH binding to NspA loop 2 mutants by ELISA. The data for wild-type (WT) NspA and the negative control (Neg. ctrl.) are the same as in panel A. In panels A and B, the data shown are from three to four measurements in two independent experiments. In some cases, the error bars are too small to be seen. (C) SDS-PAGE of *E. coli* culture supernatants containing NspA in NOMV. Panel C contains the same lanes for the molecular weight marker, negative and positive controls shown in Fig. 1A. The positions of the arrows correspond to the two species detected, as shown in Fig. 1.

Because we were primarily interested in the region of NspA that binds human FH, we first tested the impact of substitutions in NspA loops on human FH binding. Of the loop 3 mutants, D113A exhibited a 50-fold decrease in binding human FH compared with WT NspA based on the mAb concentrations needed to give an absorbance at 405 nm (A_405 nm_) of 1.0 (approximately half of the maximum value); D118A had no detectable FH binding by ELISA (>50 fold decrease) (Fig. 4A; Table 1). A statistical comparison of the A_405 nm_ values for WT and the D113A mutant at 100 µg/mL of FH gave a *P* value of 0.059 and for the D118A mutant gave *P* = 0.001 (unpaired *t*-test). Of the loop 2 mutants, D77A showed a 25-fold decrease in FH binding (*P* = 0.07 at 100 µg/mL), and K72A had no decrease (*P* = 0.75) (Fig. 4B). The mutations did not appear to alter the amounts of NspA expression or proteolytic susceptibility judging from the amount of NspA observed in *E. coli* NOMV detected by SDS-PAGE (Fig. 4C).

**TABLE 1:** Decreases in binding of human FH and antibodies to NspA loop mutants^*a*^

NspA mutant	Loop	Human FH	mAb AL-12	Polyclonal Ab
K72A	2	---^b^	---^*b*^	---
D77A	2	25 x	---	2.3 x
K79A	2	---	10 x	---
R109A	3	---	40 x	4x
D113A	3	50 x	15 x	5x
D118A	3	>50 x	>40 x	---

^*a*^ Relative to WT NspA as measured by the difference in concentration or dilution needed to obtain a threshold absorbance value by ELISA.

^*b*^ ---, no change in binding.

**Fig 5:**
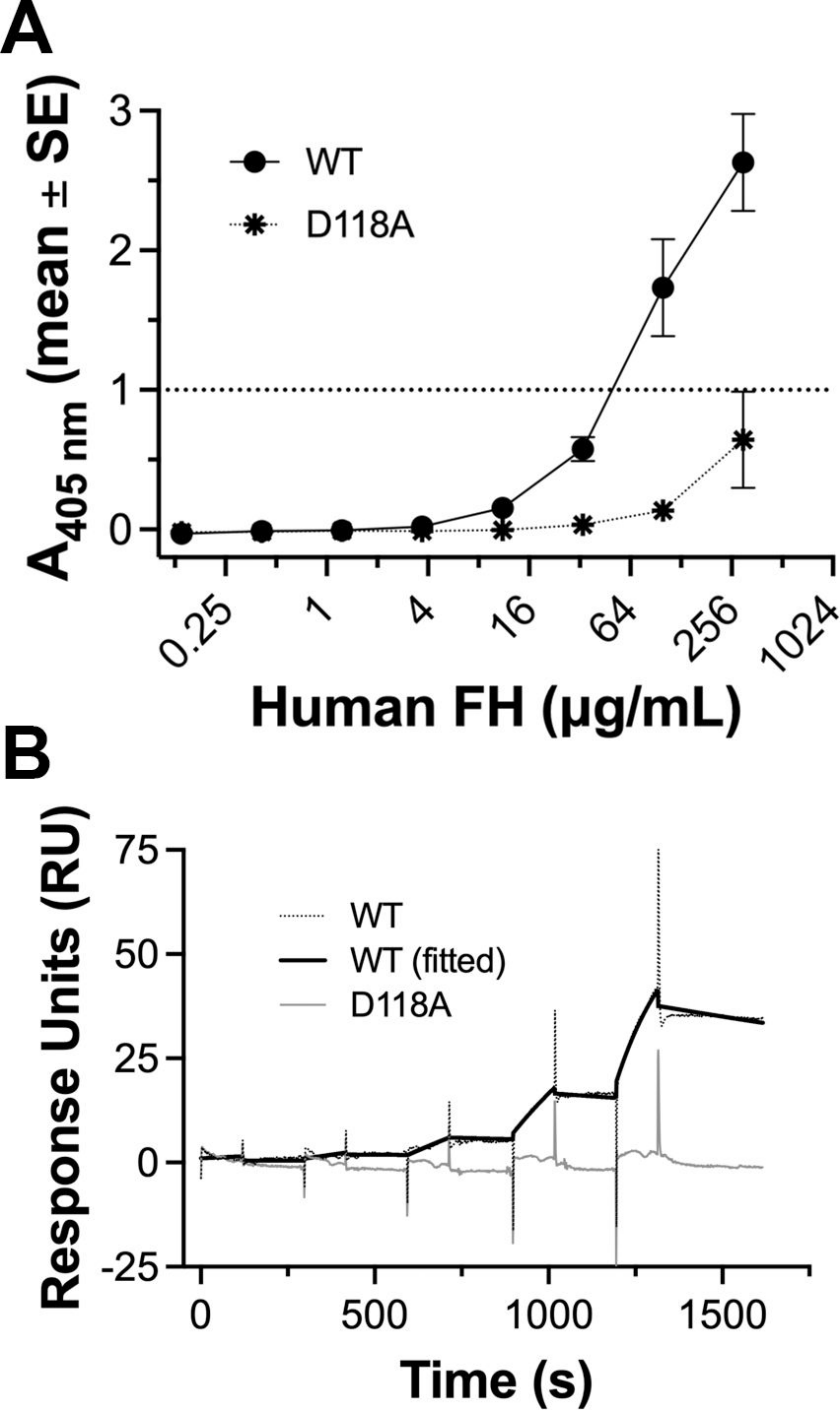
Binding of human FH to purified, refolded NspA. (A) Binding of human FH to purified, refolded NspA WT or D118A mutant by ELISA. The symbols and error bars represent the mean ± SE from quadruplicate measurements. In some cases, the error bars are too small to be seen. (B) SPR sensorgrams showing binding of purified, refolded NspA to mAb-captured human FH. Dotted black line, WT NspA (reference subtracted data); solid black line, WT NspA fitted to 1:1 binding model; solid gray line, D118A mutant (reference subtracted data).

To confirm the effects of D118A substitution, we measured the binding of human FH to purified, refolded WT and D118A mutant NspA. For these experiments, we used a closely related NspA from strain 8047 (98% identical to that from strain Su1/06), without its signal sequence. By ELISA, we observed that human FH bound to refolded WT NspA, which indicated that the protein had adopted a native-like conformation (Fig. 5A). In contrast, the refolded D118A mutant displayed a ~ tenfold decrease in binding human FH (*P* = 0.001 at 300 µg/mL). We also tested the binding of refolded NspA to antibody-captured human FH by surface plasmon resonance (SPR) (Fig. 5B). Refolded WT NspA bound to human FH with an apparent dissociation constant (*K_D_*) of 24.8 nM (Table 2). Binding of the refolded NspA D118A mutant to human FH was not detectable by SPR (Fig. 5B).

**TABLE 2:** Kinetic parameters for NspA binding to mAb-captured human FH by SPR^*a*^

NspA analyte	*k_a_* (M^−1^s^−1^)	*k_d_* (s^−1^)	*K_D_* (M)	R_max_ (RU)
WT	2.51 ± 1.76 x 10^4^	6.08 ± 4.05×10^−4^	2.48 ± 0.90 x 10^−8^	33.14 ± 19.4
D118A	---^*b*^	---	---	---

^*a*^ Data for WT NspA are the means and SD from four independent experiments; the D118A mutant was tested in two independent experiments.

^*b*^ ---, no binding detected.

### Effects of substitutions on mAb binding to NspA

**Fig 6:**
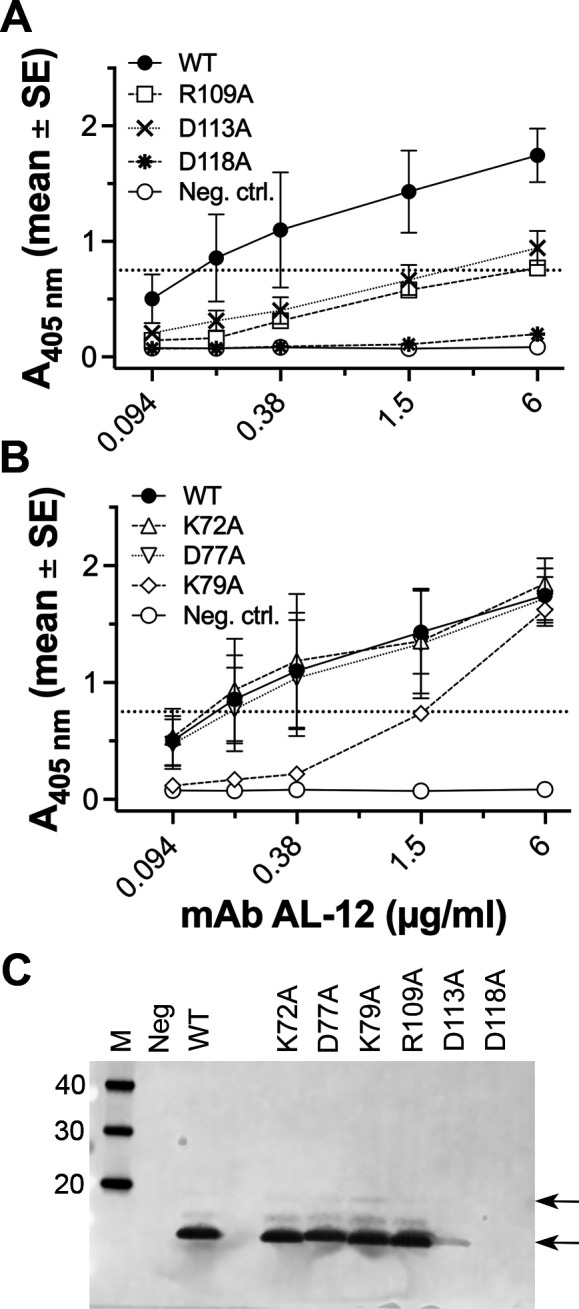
Binding of anti-NspA mAb AL-12 to NspA mutants. (A) Binding of mAb AL-12 to WT NspA and loop 3 mutants expressed in whole *E. coli* bacteria measured by ELISA. WT, wild-type NspA; Neg ctrl., *E. coli* containing the parental pUC19 plasmid lacking the NspA gene. (B) Binding of mAb AL-12 to NspA loop 2 mutants by ELISA. In panels A and B, the data shown are from two to four measurements in two independent experiments. In some cases, the error bars are too small to be seen. (C) Binding of mAb AL-12 to WT and mutant NspA expressed in whole *E. coli* lysates detected by Western blotting. Panel C contains the same lanes for the controls shown in Fig. 1C. The positions of the arrows correspond to the two species detected, as shown in Fig. 1.

Several anti-NspA mAbs recognize a predominant epitope in which natural NspA polymorphisms affect the binding of the mAbs (18). To determine whether additional amino acid residues of NspA are involved in binding of one of these mAbs, AL-12, to NspA, we tested the ability of AL-12 to bind each of the 10 NspA mutants expressed in whole *E. coli* bacteria by ELISA and Western blotting. The three substitutions in loop 3 decreased binding of mAb AL-12 to NspA by ELISA (Fig. 6A). Based on the mAb concentrations needed to give an A_405 nm_ of 0.75 (approximately half of the maximum value), the R109A and D113A mutants exhibited 40- and 15-fold decreases in mAb binding, respectively, compared with WT NspA (Table 1) (*P* = 0.049 and 0.027, respectively, at 6 µg/mL). Further, mAb AL-12 showed no detectable binding to the D118A mutant (>40 fold decrease) and was the same as binding to the negative control, which was whole *E. coli* bacteria containing a plasmid lacking NspA (*P* = 0.001 at 6 µg/mL). In contrast, neither the K72A or D77A substitutions in loop 2 affected mAb binding, whereas the K79A mutant had tenfold lower binding compared to WT (Fig. 6B). None of the four substitutions in loops 1 and 4 had an effect on mAb binding (data not shown). On Western blots of whole *E. coli* expressing recombinant NspA, the D113A mutant had decreased binding of AL-12, and D118A had no detectable binding (Fig. 6C). Although the R109A substitution decreased AL-12 binding to NspA in ELISA, there was no difference from WT NspA in Western blots, which suggests that NspA may have adopted different conformations under the conditions of the two experiments. Similar to the results from ELISA, none of the loop 2 mutants impacted mAb binding to Western blots.

### Impact of substitutions on binding of polyclonal antibodies to NspA

**Fig 7:**
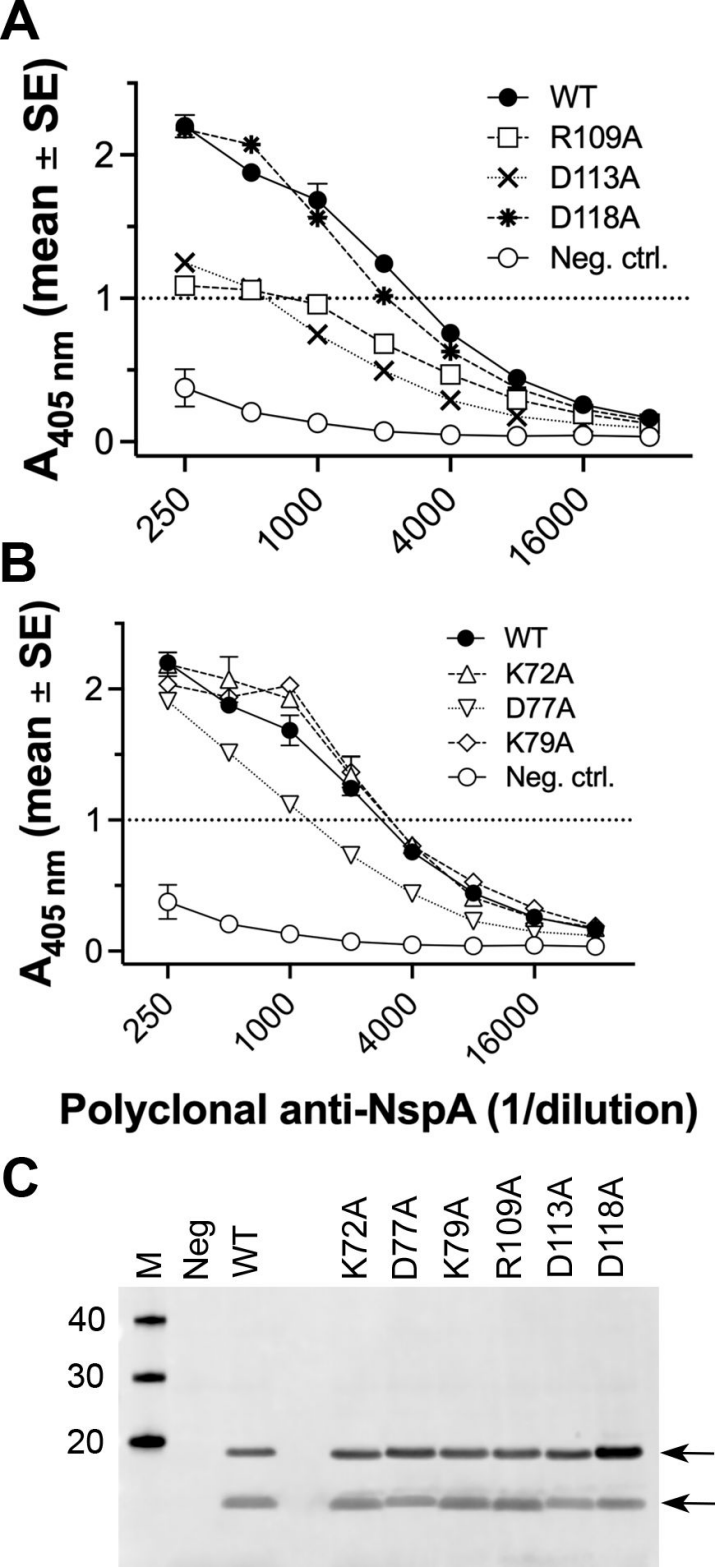
Binding of polyclonal anti-NspA antibodies to NspA mutants. (A) Binding of polyclonal anti-NspA antibodies to WT NspA and loop 3 mutants expressed in whole *E. coli* bacteria measured by ELISA. (B) Binding of polyclonal anti-NspA antibodies to loop 2 mutants by ELISA. In panels A and B, the data shown are from two to four measurements in two independent experiments. In some cases, the error bars are too small to be seen. (C) Binding of polyclonal anti-NspA antibodies to WT and mutant NspA expressed in whole *E. coli* lysates detected by Western blotting. Panel C contains the same lanes for the controls shown in Fig. 1D. The positions of the arrows correspond to the two species shown in Fig. 1.

Because NspA is likely to bear multiple epitopes, we also tested whether substitutions in the extracellular loops of NspA affected the binding of polyclonal antibodies to NspA. By ELISA, at dilutions of polyclonal antibodies needed to give an A_405 nm_ of 1.0 (approximately half of the maximum value), the R109A and D113A mutants decreased binding fourfold and fivefold, respectively (Fig. 7A; Table 1). The D118A mutant, which did not bind mAb AL-12, bound polyclonal antibodies similarly to WT NspA. Of the loop 2 mutants, D77A decreased the binding of polyclonal antibodies 2.3-fold compared to WT (Fig. 7B). By Western blotting, the NspA loop 2 and 3 mutants bound polyclonal antibodies similarly to WT NspA (Fig. 7C).

## DISCUSSION

NspA is a surface-exposed outer membrane protein that is highly conserved among meningococcal strains, and this antigen has been tested as a vaccine candidate (7, 21). Two groups independently demonstrated that anti-NspA monoclonal and/or polyclonal antibodies elicited bactericidal activity and protection against systemic meningococcal challenge in rodents (7, 8, 16, 19). Despite the success of these preclinical immunogenicity studies, purified unfolded NspA failed to induce bactericidal antibodies in a phase I clinical trial in humans (10). In retrospect, this result is not surprising since protective anti-NspA mAbs recognized conformational epitopes that are only present on NspA presented in the outer membrane or refolded under specific conditions (Fig. 1 and 6 and ref. (18)).

After the phase I study was published, NspA was identified as a second meningococcal ligand for human FH in addition to FHbp (13). This finding led our colleagues to demonstrate that the presence of human FH decreased NspA immunogenicity in human FH transgenic mice compared with WT mice (15). The effect of binding human FH by NspA on reducing immunogenicity is similar to the effect previously observed with FH on the immunogenicity of FHbp, presumably because FH can mask important epitopes (22). Together, these results suggest that a mutant NspA antigen engineered to decreased binding of human FH might also result in increased immunogenicity in the presence of human FH.

The objective of the present study was to identify NspA mutants with decreased human FH binding as a first step to develop improved NspA antigens. Our approach was to systematically test the role of each of the 10 charged residues in the four extracellular loops of NspA in binding human FH. Because it is important for the mutant vaccine antigens to retain immunogenic epitopes, we also tested the binding of anti-NspA antibodies to the NspA mutants. Previous studies showed that external loops 2 and 3 of NspA are involved in binding human FH (13) and certain anti-NspA mAbs (18). However, these studies were limited by the small number of natural amino acid sequence polymorphisms present in NspA from different Neisserial strains. In addition, because loops 1 and 4 contain only one natural polymorphism, which differs between meningococcal and gonococcal NspA, potential roles for these loops remained unclear.

**Fig 8:**
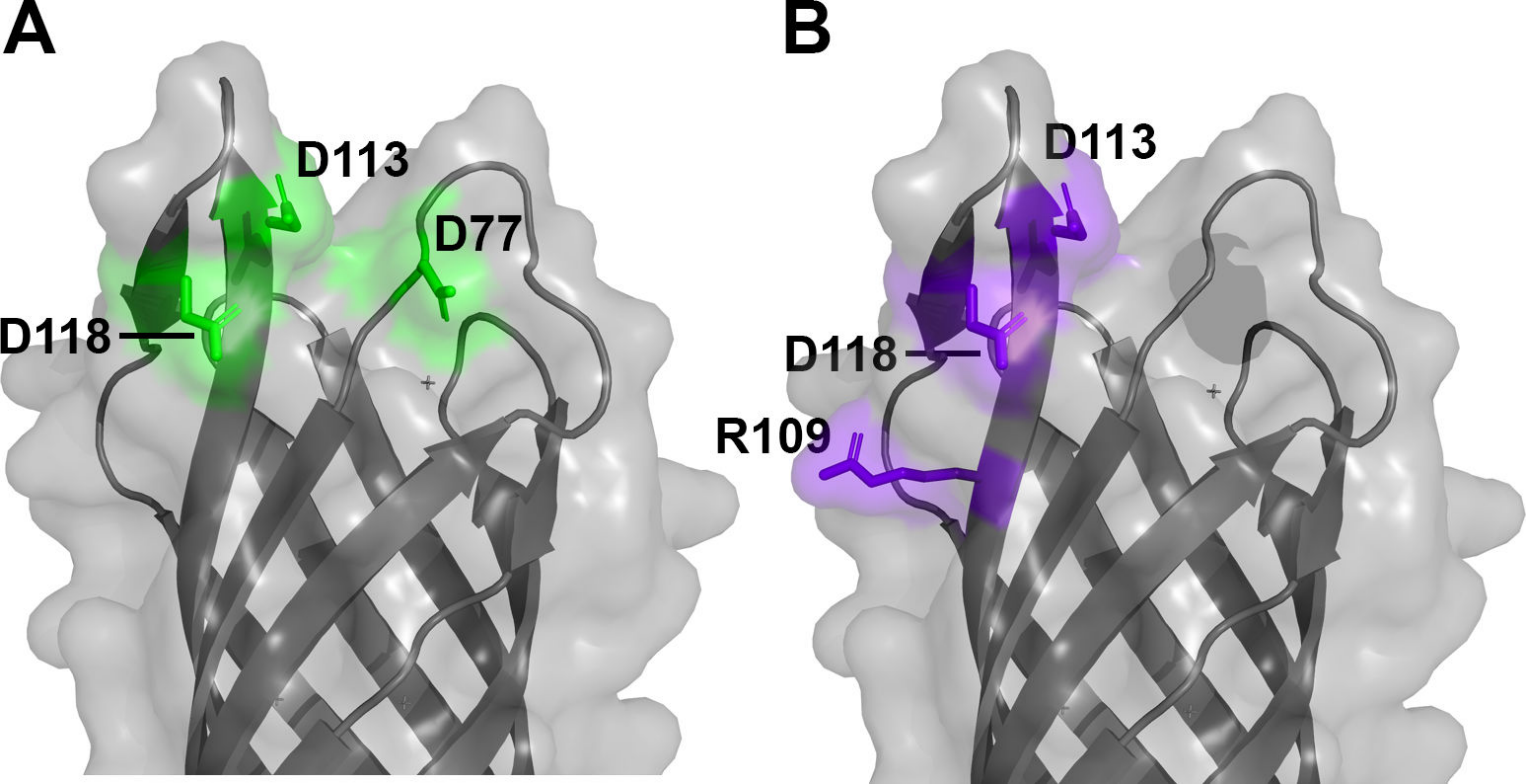
Surface-exposed regions of NspA recognized by human FH and anti-NspA mAb AL-12. (A) Residues involved in binding of human FH to NspA based on site-specific mutagenesis and binding experiments. (B) Residues implicated in binding of anti-NspA mAb AL-12. The binding sites are overlapping but distinct. Figure prepared with PyMol (The PyMOL Molecular Graphics System, Version 3.0 Schrödinger, LLC).

The most striking result of this study is that the D118 substitution exhibited little or no detectable binding of human FH. We obtained this result in ELISAs with NspA expressed in the *E. coli* outer membrane and with refolded NspA, as well as in SPR experiments with refolded NspA. Based on the crystal structure of NspA (9), residue D118 in loop 3 forms a salt bridge with K79 in loop 2. Thus, the dramatic effect of the D118A substitution on binding human FH could be attributed to direct effects on the interaction with human FH or to indirect effects of ablating this salt bridge and altering the conformation(s) of one or both of the loops. Since the K79A substitution in NspA did not affect the binding of human FH, a direct effect of the D118A substitution on FH binding appears more likely. We also found that the D77A and D113A substitutions in NspA each decreased the binding of FH ~25 and 50-fold, respectively. When we mapped the three residues that were involved in binding human FH onto the structure of NspA, the residues constituted a surface-exposed region on a face of the beta-barrel that was oriented to the periphery of the barrel (Fig. 8A). By ELISA, anti-NspA mAb AL-12 inhibited binding of human FH, which suggested that their binding sites overlapped. Using the same set of site-specific mutants, we found that NspA residues D113, R109, and D118 are involved in binding anti-NspA mAb AL-12. When we mapped the residues implicated in forming the epitope recognized by mAb AL-12 onto the surface of NspA, they formed a contiguous surface that overlapped with the FH binding site (Fig. 8B).

As described in the *Introduction*, natural sequence polymorphisms in meningococcal NspA have been used to determine the sites recognized by anti-NspA mAbs (18). For example, a glutamine (Q) residue at position 73 in loop 2 of NspA is present in most gonococcal strains and absent from most meningococcal strains. When inserted into meningococcal NspA, Q73 decreased binding of human FH (14). This residue is adjacent to K72, which did not affect FH binding when mutated to A in the present study. Therefore, it is likely that the length of loop 2 or positioning of residues other than K72 impacts binding of human FH to NspA. In loop 3 of meningococcal NspA, valine (V) 112 and D113 corresponded with A and histidine (H) in gonococcal NspA. Changing these two residues from VD to AH decreased FH binding in a meningococcal NspA background, and the converse substitutions increased FH binding in a gonococcal NspA sequence (14). In the present study, substitution of D113 alone decreased FH binding when replaced by A (Fig. 4A) or H (data not shown); the V112A substitution had no effect on FH binding.

In a published study, insertion of Q73 in loop 2 of meningococcal NspA greatly decreased binding of mAbs AL-12 and 14C7 (18). Further, substitution of leucine (L) 114 and glycine (G) 115 in loop 3 of a reactive NspA sequence (strain 8047) to the corresponding residues, phenylalanine (F) and asparagine (N), from a nonreactive sequence (strain MCH88) also decreased binding of the two mAbs (18). The nonpolar side chains of L114 and G115 in NspA from the strain with high mAb binding suggest that either hydrophobic interactions are important for mAb binding or the substitution of bulkier side chains interferes with mAb binding. Further, the observations that natural polymorphisms and targeted substitutions in loops 2 and 3 of NspA affect binding of human FH (14) and anti-NspA mAbs (18) suggest that this region is a binding hotspot. The functional role of the polymorphisms remains unclear but might play a role in conjunction with NspA expression to regulate the amount of FH bound to the bacterial surface.

In summary, we have identified several charged residues in loops 2 and 3 of NspA that are involved in binding to human FH and mAb AL-12, which recognizes a conformational epitope. As described above, the binding sites overlap but are distinct (Fig. 8). This knowledge could be valuable in the development of a mutated NspA as a vaccine antigen. Although D77A decreases binding of human FH without affecting mAb binding, it is not known whether the approximate 25-fold decrease will be sufficient to maximize the protective antibody responses (23) given the high concentration of FH in human serum, ~230 to 280 µg/mL (22, 24, 25). Larger decreases in binding human FH might be achieved, for example, by introducing larger side chains at position 77 that would introduce a charge repulsion or steric hindrance for binding human FH but not disrupt the binding of bactericidal mAbs such as AL-12. Such NspA mutants with sufficient decreases in binding of human FH, and that retain mAb binding, could be used as recombinant NspA antigens or expressed in NOMV as vaccine antigens against invasive meningococcal disease. Finally, the high sequence conservation of NspA among gonococcal strains and its presence in the outer membrane have led NspA to be proposed as a vaccine candidate against gonorrhea (26). Further, its role as a ligand for human FH (13, 14) warrants further investigation as a gonococcal vaccine antigen.

## MATERIALS AND METHODS

### Site-specific mutagenesis of NspA

Site-specific mutants of NspA were generated using the Q5 Site-directed Mutagenesis Kit (New England Biolabs). The plasmid template for mutagenesis was pEHNspA, a pUC19-based plasmid containing the NspA gene from strain Su1/06 with a PorA/NadA hybrid promoter, which replaces the homopolymeric nucleotide tract in the PorA promoter with the corresponding sequence from NadA (15). Primers with the desired nucleotide changes in the NspA gene are listed in Table 3. The sequences of the mutant NspA genes were determined by Sanger sequencing (Genewiz).

**TABLE 3:** Oligonucleotide primers used to generate NspA site-directed mutants

Primer name^*a*^	Sequence^*b*^
L1-K44A –F L1-K44A – R	TCTGCCGCAGGCTTCAG ACCTAAAGAGCTTGAGGCTTTTGC
L2-K72A – F L2-K72A – R	TATGCAGCCCCATCCACC GTTTTTGTAGCGCGTGTAATCGAC
L2-D77A – F L2-D77A – R	ACCGCTTTCAAACTTTACAGCATC GGATGGGGCTTTATAGTTTTTGTAG
L2-K79A – F L2-K79A – R	TTCGCACTTTACAGCATCGGC ATCGGTGGATGGGGCTTTATAG
L3-R109A – F L3-R109A – R	GAGCCTCAACGCCGCCTCCGTCGAC AAGCGCGCGCCGAGATAC
L3-D113A – F L3-D113A – R	TCCGTCGCCTTGGGCGG GGCGCGGTTGAGGCTCAA
L3-D118A – F L3-D118A – R	GGCAGCGCCAGCTTCAGC GCCCAAGTCGACGGAGGC
L4-K154A – F L4-K154A – R	ATCGGCGCAGTCAACACTG GTAGTTGTAGCGGTAGCCGG
L4-K159A – F L4-K159A – R	ACTGTCGCAAACGTCCGTTC GTTGACTTTGCCGATGTAGTTG
L4-R162A – F L4-R162A – R	GTCGCTTCCGGCGAACTG GTTTTTGACAGTGTTGACTTTGCC

^*a*^ L1 through L4 correspond to the four extracellular loops of NspA. The numbering of the amino acids is based on the full-length sequence of NspA including the signal sequence following a previous convention (18). F, forward; R, reverse.

^*b*^ Nucleotide sequence (5' to 3') shows mutated bases in forward primers underlined.

### Expression of NspA in *E. coli*

The NspA gene including its signal sequence from *N. meningitidis* strain Su1/06 was expressed from plasmid pEHNspA (15) in *E. coli* BL21(DE3) (New England Biolabs). The parental pUC19 plasmid was used as a negative control. The bacterial strains were grown in lysogeny broth (LB) containing 50 µg/mL ampicillin at 37°C for 18 hours. Whole bacteria, culture supernatants containing NOMV and bacterial lysates were prepared as follows. Overnight cultures were centrifuged at 4,000 x *g* for 10 minutes at room temperature (RT) to collect the bacteria, which were resuspended in phosphate-buffered saline (PBS). Different samples of whole bacteria used for ELISA and Western blotting were normalized to the same optical density measured at 600 nm (OD_600nm_). Culture supernatants were concentrated tenfold using 10 kDa molecular weight cut off centrifugal concentrators (Corning Spin-X UF). To prepare whole bacterial lysates for Western blotting, bacteria were suspended in in RIPA buffer (25 mM Tris, 150 mM NaCl, 1% nonidet P-40, 1% sodium deoxycholate, 0.1% SDS, pH 7.6) containing EDTA-free protease inhibitor cocktail (Roche), frozen, thawed, and incubated at 37°C for 1 hour. The total protein concentrations of the bacterial lysates were determined by bicinchoninic acid (BCA) assay (ThermoScientific).

### NspA expression, purification, and refolding

The NspA gene from strain 8047 lacking a signal sequence was expressed from plasmid pET28-NspA in *E. coli* BL21(DE3), which encoded a hexahistidine- (His_6_-) tag on the carboxyl terminus of NspA. Cultures were grown in terrific broth (Sigma) containing 0.3% ethanol and 50 µg/mL kanamycin. NspA expression was induced at an OD_600 nm_ of 0.8 to 0.9 with 0.2 mM isopropylthiogalactoside (IPTG) for 4 hours at 37°C. Expression of NspA was monitored by SDS-PAGE using 4%–12% NuPAGE Bis-Tris gels (ThermoScientific) run in 2-(N-morpholino) ethanesulfonic acid (MES) buffer (ThermoScientific) at 200 V for 45 minutes and stained with Coomassie Brilliant blue R-250 (Sigma) in 45% (vol/vol) methanol and 10% (vol/vol) acetic acid (Fisher Scientific).

For purification, bacteria were lysed by freeze–thaw treatment in the presence of 0.5 mg/mL chicken egg white lysozyme (Sigma), followed by sonication. Inclusion bodies containing NspA were obtained by centrifugation at 16,000 x *g* for 30 minutes. The pellet containing inclusion bodies was suspended in 50 mM HEPES, 1.5% lauryldimethylamine oxide (LDAO), pH 8.0, for 1 hour at room temperature (RT) and washed twice with 50 mM 4-(2-hydroxyethyl)−1-piperazineethanesulfonic acid (HEPES), pH 8.0. The inclusion bodies were then solubilized in 10 mM HEPES, 1 mM ethylenediaminetetraacetic acid (EDTA), 8 M urea, pH 8.0, and centrifuged at 14,000 x *g* for 30 minutes to remove insoluble debris. Solubilized NspA-His_6_ was applied to an Ni^2+^ chelating column (HisTrap HP 5 mL; GE Life Sciences) and eluted with a gradient of 20 to 250 mM imidazole in 20 mM HEPES, 8 M urea, 0.5 M NaCl, pH 8.0. The purified protein was dialyzed in the same buffer without imidazole, and the concentration was determined by BCA assay.

Purified NspA in 8 M urea was refolded using a preparative membrane protein folding column MFC15 (ProFoldin). Refolded NspA was further purified on a Superdex 200 HiLoad 26/60 column in 20 mM HEPES, 1 mM dodecyl maltoside, 2 mM EDTA, 50 mM NaCl, pH 8.5 to remove aggregates. The concentration of the refolded protein was determined by BCA assay.

### Generation of polyclonal anti-NspA antibodies

We used purified, unfolded NspA from strain 8047 for the production of polyclonal antibodies in mice. NspA in 8 M urea was dialyzed overnight in 2 M urea, 10 mM L-histidine 0.15 M NaCl, pH 6.5. Five-week-old CD-1 female mice were immunized intraperitoneally with three doses of purified NspA-His_6_ protein with Titermax (Sigma) as the adjuvant. The doses (10 µg each) were administered at 3-week intervals. Whole blood was collected from the immunized mice by cardiac puncture 2 weeks after the third dose, and the sera were separated and stored at −70°C prior to assay. Individual serum samples were tested for anti-NspA reactivity, and serum pools were used to detect NspA in whole *E. coli* bacteria or culture supernatants containing NOMV by ELISA and Western blotting.

### Western blotting

In Western blotting experiments, whole *E. coli* lysates, concentrated culture supernatants, or purified recombinant NspA were separated by SDS-PAGE as described above and were transferred to polyvinylidene fluoride (PVDF) membranes (Millipore Immobilon FL) using an XCell II blot module (Invitrogen). The membranes were blocked with 5% skimmed milk (Carnation) in PBST overnight at 4°C. The membranes were incubated with anti-NspA polyclonal antisera produced as described above (1:2,500 dilution) or mAb AL-12 (0.8 µg/mL) for 2 hours at RT. Bound antibodies were detected with goat anti-mouse IgG antibody conjugated to an infrared dye (Li-Cor IRDye800; 1:15,000 dilution) for 1 hour at RT. Between each incubation step, the membranes were washed thrice in PBST (PBS + 0.1% Tween-20). The blots were imaged on an infrared scanner (Li-Cor Odyssey) at 800 nm.

### Human FH

Purified human FH was obtained from a commercial source (Complement Technologies). In some experiments, we used FH purified from human serum or human FH transgenic rat serum by affinity chromatography with meningococcal Factor H binding protein (FHbp) amine coupled to a column (GE Life Sciences HiTrap NHS 5 mL).

### FH ELISA

*E. coli* BL21(DE3) expressing NspA with a signal sequence were tested for binding of human FH by ELISA. The wells of microtiter plates (Nunc 96-well, flat-bottom plates) were coated with poly-L-lysine (Sigma) by incubation of a 0.1 µg/mL solution (50 µL/well) for 30 minutes at RT. The wells of the plates were washed thrice with PBS and then incubated with whole *E. coli* bacteria (100 µL/well) overnight at 4°C. The next day, the wells were washed as above, and nonspecific binding was blocked with PBST containing 1% skimmed milk and 2% bovine serum albumin (BSA; Equitech) for 90 minutes at RT. The wells were incubated with purified human FH (twofold serial dilutions starting at 200 µg/mL in PBST containing 0.1% skimmed milk and 0.2% BSA) for 2 hours at RT with gentle agitation. Bound FH was detected with sheep anti-human FH polyclonal antibody (Abcam; 1:7,000), which was detected with donkey anti-sheep IgG antibody conjugated to alkaline phosphatase (AP) (Sigma, 1:10,000 dilution) for 1 hour at RT. Between each incubation step, the wells were washed thrice in PBST. Para-nitrophenyl phosphate (PNPP; Sigma, 1 mg/mL) in substrate buffer (50 mM sodium carbonate, 1 mM magnesium chloride, pH 9.8) was added to the wells, incubated for 30 minutes at RT, and the A_405 nm_ was read in a plate reader (Molecular Devices SpectraMax 190). For all of the ELISA experiments, data are shown from two independent experiments (total of two to four replicates).

To test binding of human FH to purified refolded NspA, the wells of microtiter plates (Nunc Immulon 2HB) were coated with 100 µL/well of 5 µg /mL of refolded NspA in PBS overnight at 4°C. Nonspecific binding to the wells was blocked with PBST containing 2% BSA for 90 minutes at RT. Bound human FH was detected as described above.

### Anti-NspA antibody ELISA

For whole bacterial cell ELISA, the wells of microtiter plates were coated with poly-L-lysine followed by bacterial cells as described above. The wells were washed thrice with PBST, and nonspecific binding was blocked with 5% skimmed milk in PBS for 90 minutes at RT. The wells were incubated with mAb AL-12 (twofold or threefold serial dilutions starting at 6 µg/mL) or anti-NspA polyclonal antisera (twofold or threefold serial dilutions starting at 1:250) in PBS containing 0.5% skimmed milk for 2 hours. Bound IgG antibodies were detected with a goat anti-mouse IgG antibody conjugated to AP (Sigma, 1:5,000 dilution in PBS containing 0.5% skimmed milk) for 1 hour at RT. AP substrate addition and colorimetric detection were performed as described above.

To detect anti-NspA antibody binding to purified refolded NspA, the wells of a microtiter plate (Nunc Immulon 2HB) were coated with 5 µg /mL of refolded NspA (100 µL/well) in PBS overnight at 4°C. The wells were blocked with PBS containing 2% BSA, washed with PBST, and then incubated with anti-NspA mAb AL-12 mAb or mouse anti-NspA polyclonal antisera. Bound mouse IgG antibodies were detected as described above.

### Inhibition ELISA

The ability of mAb AL-12 to compete with human FH for binding to NspA was tested by inhibition ELISA. In this assay, serial dilutions of mAb AL-12 were used to inhibit binding of a fixed concentration of human FH (100 µg/ml) to purified refolded NspA. mAb AL-12 and human FH were pre-mixed and then added to the wells of the ELISA plate containing refolded NspA. Bound human FH was detected with rabbit anti-human FH polyclonal antibody, which was produced commercially (Sino) and diluted 1:5,000 in PBST containing 0.2% BSA, followed by a goat anti-rabbit IgG antibody conjugated to AP (Sigma; 1:5,000 dilution in the same buffer).

### Kinetic analysis of NspA binding to human FH

The kinetics of NspA and NspA mutants binding to human FH were determined by SPR using a Biacore X100 Plus instrument (GE Life Sciences). About 5,500 response units (RU) of anti-human FH mAb (Q254; Quidel) were immobilized on a CM5 sensor chip (Cytiva Life Sciences) using a Biacore Amine Coupling Kit (GE Life Sciences). Single-cycle kinetic experiments were conducted by capturing purified human FH (5 µg/mL, 3 minutes at 30 µL/minutes) with the immobilized anti-FH mAb then injecting different concentrations of purified, refolded NspA in running buffer (10 mM HEPES, 150 mM NaCl, 3 mM EDTA, 0.005% surfactant P-20, pH 7.4). Five serial 3.16-fold dilutions of NspA starting at 10 µM were each injected for 2 minutes, followed by a 5-minute dissociation. The biosensor chip surface was regenerated with 10 mM glycine, pH 1.7, with a contact time of 1 minute. Kinetic parameters (*k_a_*, *k_d,_* and *K_D_*) were determined using BIAevaluation X100 software version 2.0.2 (Cytiva Life Sciences) and a 1:1 binding model.
